# 1,4-Bis(carb­oxy­meth­yl)piperazine-1,4-diium bis­(dihydrogen phosphate) dihydrate

**DOI:** 10.1107/S1600536810036342

**Published:** 2010-09-15

**Authors:** Lin Cheng, Li-Min Zhang, Jian-Quan Wang

**Affiliations:** a Department of Chemistry and Chemical Engineering, Southeast University, Nanjing 211189, People’s Republic of China

## Abstract

In the title salt, C_8_H_16_N_2_O_4_^2+^·2H_2_PO_4_^−^·2H_2_O, the piperazine ring is located around an inversion center and adopts a chair conformation. The dihydrogen phosphate anions and free water mol­ecules are linked *via* O—H⋯O hydrogen bonds into two-dimensional hydrogen-bonding layers, which are further connected through O—H⋯O and N—H⋯O hydrogen bonds involving the protonated piperazine into a three-dimensional supra­molecular network.

## Related literature

For related structures, see: Yang *et al.* (2008 ▶). For potential applications of optical, electrical, magnetic and microporous materials, see: Evans & Lin (2002 ▶); Zhang & Chen (2006 ▶).
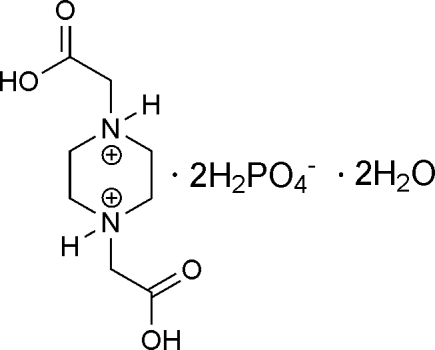


## Experimental

### 

#### Crystal data

C_8_H_16_N_2_O_4_^2+^·2H_2_PO_4_^−^·2H_2_O
*M* *_r_* = 434.23
Monoclinic, 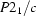

*a* = 8.716 (3) Å
*b* = 8.992 (3) Å
*c* = 12.991 (4) Å
β = 123.310 (17)°
*V* = 850.9 (5) Å^3^
*Z* = 2
Mo *K*α radiation
μ = 0.33 mm^−1^
*T* = 120 K
0.54 × 0.44 × 0.41 mm

#### Data collection

Bruker SMART APEX CCD diffractometer
Absorption correction: multi-scan (*SADABS*; Sheldrick, 2003 ▶) *T*_min_ = 0.840, *T*_max_ = 0.875
3998 measured reflections
1668 independent reflections
1552 reflections with *I* > 2σ(*I*)
*R*_int_ = 0.022

#### Refinement

*R*[*F* ^2^ > 2σ(*F* ^2^)] = 0.035
*wR*(*F* ^2^) = 0.098
*S* = 1.09
1668 reflections
121 parameters
H-atom parameters constrained
Δρ_max_ = 0.49 e Å^−3^
Δρ_min_ = −0.54 e Å^−3^

### 

Data collection: *SMART* (Bruker, 2000 ▶); cell refinement: *SAINT* (Bruker, 2000 ▶); data reduction: *SAINT*; program(s) used to solve structure: *SHELXTL* (Sheldrick, 2008 ▶); program(s) used to refine structure: *SHELXL97* (Sheldrick, 2008 ▶); molecular graphics: *ORTEPIII* (Burnett & Johnson, 1996 ▶), *ORTEP-3 for Windows* (Farrugia, 1997 ▶) and *PLATON* (Spek, 2009 ▶); software used to prepare material for publication: *SHELXL97*.

## Supplementary Material

Crystal structure: contains datablocks global, I. DOI: 10.1107/S1600536810036342/dn2598sup1.cif

Structure factors: contains datablocks I. DOI: 10.1107/S1600536810036342/dn2598Isup2.hkl

Additional supplementary materials:  crystallographic information; 3D view; checkCIF report

## Figures and Tables

**Table 1:** Hydrogen-bond geometry (Å, °)

*D*—H⋯*A*	*D*—H	H⋯*A*	*D*⋯*A*	*D*—H⋯*A*
O2—H2⋯O3	0.84	1.69	2.5324 (18)	177
N1—H1⋯O5^i^	0.93	1.74	2.658 (2)	170
O4—H4⋯O1*W*	0.84	1.74	2.5729 (19)	172
O6—H6⋯O5^ii^	0.84	1.74	2.5700 (17)	169
O1*W*—H1*WA*⋯O6^iii^	0.85	2.08	2.868 (2)	155
O1*W*—H1*WB*⋯O3^iv^	0.85	2.02	2.8633 (19)	169

Symmetry codes: (i) 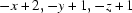; (ii) 
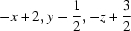
; (iii) 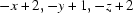; (iv) 
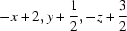
.

**Fig. 1.**
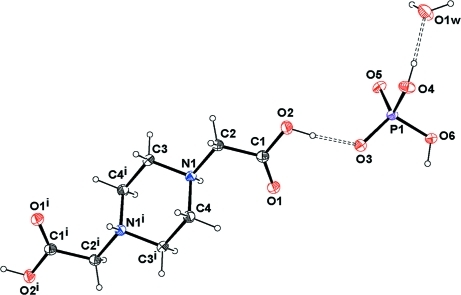
Molecular view of compound (I) with the atom-labelling scheme. Displacement ellipsoids are drawn at the 30% probability level. H atoms are represented as small spheres of arbitrary radii. Hydrogen bonds are shown as dashed lines.

**Fig. 2.**
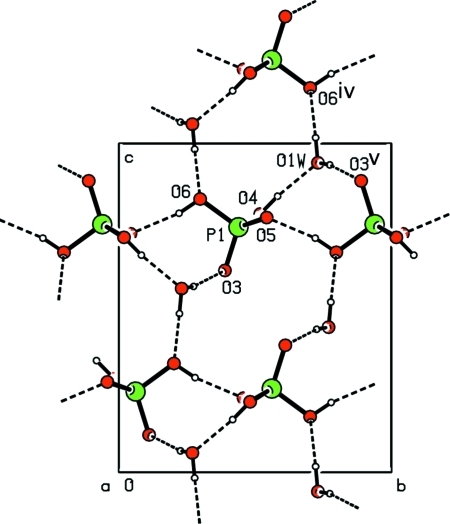
Partial packing view of compound ( I ), showing the two-dimensional network formed by the hydrogen bonds showed as dashed lines, involving the dihydrogen phosphate and the water molecules. [Symmetry codes:(iv) -x+2, -y+1,-z+2; (v) -x+2, y+1/2, -z+3/2]

## supplementary crystallographic information

### Comment

Recent years have witnessed an explosion of great interest in hybrid organic-inorganic framework solids not only for their intriguing architectures and topologies, but also for their potential applications in optical, electrical, magnetic, and microporous materials (Evans *et al.* 2002; Zhang *et al.* 2006). We have synthesized two series of hybrid organic-inorganic frameworks with 1,4-piperazinediacetic acid and lanthanide sulfates (Yang *et al.* 2008). Our aim is to obtain similar hybrid solids by using phosphates instead of sulfates. However, we fail to synthesize the aimed compounds and obtain a three-dimensional supramolecular network, C_8_H_24_N_2_O_14_P_2_ (1.2H_2_PO_4_.2H_2_O).

The title compound, is a dihydrogen phosphate, in which 1 is a protonated piperazine derivative and the piperazine ring located around inversion center adopts chair conformation (Fig. 1). The asymmetric unit of the title compound, contains half a protonated 1,4-piperazinediacetic acid, a dihydrogen phosphate and a free water molecule. In the carboxylates of protonated 1,4-piperazinediacetic acid, the distance of the C=O bonds is 1.209 (2) Å, which is shorter than those of C—O bond (1.311 (2) Å) and considered to have full double-bond character.

In the compound, the dihydrogen phosphates and free water molecules are linked to each other, *via* O—H···O hydrogen bonds into a two-dimensional hydrogen bonding layers (Table 1, Fig. 2), which are further connected through O—H···O and N—H···O hydrogen bonds involving the protonated 1,4-piperazinediacetic acid into a three-dimensional supramolecular network.

### Experimental

A mixture of H_2_pda.2H_2_O (0.024 g, 0.1 mmol), Nd_2_O_3_ (0.034 g, 0.1 mmol), H_3_PO_4_ (0.1 ml), and water (6 ml) were heated ina 15 ml Teflon-lined vessel at 160 ° for 3 days, followed by slow cooling (5 ° h^-1^) to room temperature. After filtration, colorless block crystals were collected and dried in air (0.025 g, yield *ca* 57% based on H_2_pda).

### Refinement

All H atoms attached to C, N and O(hydroxyl) atoms were fixed geometrically and treated as riding with C—H = 0.99 Å (methylene), N—H = 0.93 Å and O—H= 0.84 Å with U_iso_(H) = 1.2U_eq_(C or N) or U_iso_(H) = 1.5U_eq_(O). H atoms of water molecule were located in difference Fourier maps and included in the subsequent refinement using restraints (O-H= 0.85 (1)Å and H···H= 1.40 (2)Å) with U_iso_(H) = 1.5U_eq_(O). In the last cycle of refinement, they were treated as riding on their parent O atom.

### Crystal data

**Table:**

C_8_H_16_N_2_O_4_^2+^·2H_2_PO_4_^−^·2H_2_O	*F*(000) = 456
*M**_r_* = 434.23	*D*_x_ = 1.695 Mg m^−^^3^
Monoclinic, *P*2_1_/*c*	Mo *K*α radiation, λ = 0.71073 Å
Hall symbol: -P 2ybc	Cell parameters from 782 reflections
*a* = 8.716 (3) Å	θ = 2.4–28.0°
*b* = 8.992 (3) Å	µ = 0.33 mm^−^^1^
*c* = 12.991 (4) Å	*T* = 120 K
β = 123.310 (17)°	Block, colourless
*V* = 850.9 (5) Å^3^	0.54 × 0.44 × 0.41 mm
*Z* = 2	

### Data collection

**Table:**

Bruker SMART APEX CCD diffractometer	1668 independent reflections
Radiation source: fine-focus sealed tube	1552 reflections with *I* > 2σ(*I*)
graphite	*R*_int_ = 0.022
φ and ω scan	θ_max_ = 26.0°, θ_min_ = 2.9°
Absorption correction: multi-scan (*SADABS*; Sheldrick, 2003)	*h* = −10→8
*T*_min_ = 0.840, *T*_max_ = 0.875	*k* = −11→10
3998 measured reflections	*l* = −10→16

### Refinement

**Table:**

Refinement on *F*^2^	Primary atom site location: structure-invariant direct methods
Least-squares matrix: full	Secondary atom site location: difference Fourier map
*R*[*F*^2^ > 2σ(*F*^2^)] = 0.035	Hydrogen site location: inferred from neighbouring sites
*wR*(*F*^2^) = 0.098	H-atom parameters constrained
*S* = 1.09	*w* = 1/[σ^2^(*F*_o_^2^) + (0.0583*P*)^2^ + 0.4175*P*] where *P* = (*F*_o_^2^ + 2*F*_c_^2^)/3
1668 reflections	(Δ/σ)_max_ < 0.001
121 parameters	Δρ_max_ = 0.49 e Å^−^^3^
0 restraints	Δρ_min_ = −0.54 e Å^−^^3^

### Special details

**Table:**

Geometry. All esds (except the esd in the dihedral angle between two l.s. planes) are estimated using the full covariance matrix. The cell esds are taken into account individually in the estimation of esds in distances, angles and torsion angles; correlations between esds in cell parameters are only used when they are defined by crystal symmetry. An approximate (isotropic) treatment of cell esds is used for estimating esds involving l.s. planes.
Refinement. Refinement of *F*^2^ against ALL reflections. The weighted *R*-factor *wR* and goodness of fit *S* are based on *F*^2^, conventional *R*-factors *R* are based on *F*, with *F* set to zero for negative *F*^2^. The threshold expression of *F*^2^ > σ(*F*^2^) is used only for calculating *R*-factors(gt) *etc*. and is not relevant to the choice of reflections for refinement. *R*-factors based on *F*^2^ are statistically about twice as large as those based on *F*, and *R*- factors based on ALL data will be even larger.

### Fractional atomic coordinates and isotropic or equivalent isotropic displacement parameters (Å^2^)

**Table:**

	*x*	*y*	*z*	*U*_iso_*/*U*_eq_	
O1	0.69897 (17)	0.37509 (13)	0.33962 (11)	0.0187 (3)	
O2	0.64019 (19)	0.56082 (13)	0.42897 (12)	0.0213 (3)	
H2	0.6898	0.5039	0.4903	0.032*	
N1	0.57511 (19)	0.53648 (15)	0.12844 (13)	0.0131 (3)	
H1	0.6924	0.4981	0.1622	0.016*	
C1	0.6431 (2)	0.49837 (19)	0.33891 (15)	0.0156 (4)	
C2	0.5654 (2)	0.60289 (19)	0.22984 (15)	0.0158 (3)	
H2A	0.4360	0.6257	0.1991	0.019*	
H2B	0.6353	0.6973	0.2566	0.019*	
C3	0.5428 (2)	0.65335 (18)	0.03654 (15)	0.0159 (3)	
H3A	0.6332	0.7346	0.0785	0.019*	
H3B	0.4184	0.6958	−0.0006	0.019*	
C4	0.4392 (2)	0.41261 (19)	0.06352 (16)	0.0163 (4)	
H4A	0.3131	0.4513	0.0268	0.020*	
H4B	0.4608	0.3343	0.1237	0.020*	
P1	0.92100 (6)	0.43880 (4)	0.74544 (4)	0.01286 (17)	
O3	0.79926 (16)	0.38920 (13)	0.61380 (11)	0.0174 (3)	
O4	0.80098 (16)	0.51844 (14)	0.78556 (12)	0.0200 (3)	
H4	0.8650	0.5820	0.8400	0.030*	
O5	1.07757 (15)	0.54245 (12)	0.77465 (11)	0.0160 (3)	
O6	1.00826 (16)	0.29822 (13)	0.83100 (11)	0.0172 (3)	
H6	0.9690	0.2207	0.7879	0.026*	
O1W	0.9695 (2)	0.73039 (16)	0.94132 (13)	0.0289 (3)	
H1WA	1.0120	0.7221	1.0176	0.043*	
H1WB	1.0505	0.7740	0.9349	0.043*	

### Atomic displacement parameters (Å^2^)

**Table:**

	*U*^11^	*U*^22^	*U*^33^	*U*^12^	*U*^13^	*U*^23^
O1	0.0218 (6)	0.0160 (6)	0.0182 (6)	0.0027 (5)	0.0109 (5)	0.0009 (5)
O2	0.0306 (8)	0.0185 (6)	0.0159 (6)	0.0063 (5)	0.0134 (6)	0.0025 (5)
N1	0.0134 (7)	0.0124 (6)	0.0139 (7)	−0.0002 (5)	0.0078 (6)	0.0002 (5)
C1	0.0140 (8)	0.0159 (8)	0.0163 (8)	−0.0012 (6)	0.0080 (7)	−0.0011 (6)
C2	0.0187 (8)	0.0139 (8)	0.0160 (8)	0.0018 (6)	0.0103 (7)	−0.0006 (6)
C3	0.0197 (8)	0.0118 (7)	0.0162 (8)	−0.0006 (6)	0.0098 (7)	0.0013 (6)
C4	0.0170 (8)	0.0159 (8)	0.0150 (8)	−0.0041 (6)	0.0082 (7)	−0.0003 (6)
P1	0.0137 (3)	0.0112 (3)	0.0146 (3)	−0.00065 (14)	0.0084 (2)	−0.00046 (14)
O3	0.0194 (6)	0.0157 (6)	0.0149 (6)	−0.0001 (5)	0.0081 (5)	−0.0004 (5)
O4	0.0180 (6)	0.0201 (6)	0.0253 (7)	−0.0030 (5)	0.0142 (6)	−0.0076 (5)
O5	0.0141 (6)	0.0120 (6)	0.0230 (7)	0.0003 (4)	0.0109 (5)	0.0017 (5)
O6	0.0226 (6)	0.0115 (6)	0.0154 (6)	−0.0025 (5)	0.0092 (5)	−0.0014 (4)
O1W	0.0355 (8)	0.0347 (8)	0.0219 (7)	−0.0153 (6)	0.0192 (6)	−0.0104 (6)

### Geometric parameters (Å, °)

**Table:**

O1—C1	1.209 (2)	C3—H3B	0.9900
O2—C1	1.311 (2)	C4—C3^i^	1.513 (2)
O2—H2	0.8400	C4—H4A	0.9900
N1—C2	1.490 (2)	C4—H4B	0.9900
N1—C3	1.497 (2)	P1—O3	1.5022 (13)
N1—C4	1.503 (2)	P1—O5	1.5186 (12)
N1—H1	0.9300	P1—O4	1.5740 (12)
C1—C2	1.515 (2)	P1—O6	1.5759 (13)
C2—H2A	0.9900	O4—H4	0.8400
C2—H2B	0.9900	O6—H6	0.8400
C3—C4^i^	1.513 (2)	O1W—H1WA	0.8499
C3—H3A	0.9900	O1W—H1WB	0.8505
			
C1—O2—H2	109.5	N1—C3—H3B	109.6
C2—N1—C3	110.29 (12)	C4^i^—C3—H3B	109.6
C2—N1—C4	112.53 (13)	H3A—C3—H3B	108.1
C3—N1—C4	109.14 (13)	N1—C4—C3^i^	110.54 (13)
C2—N1—H1	108.3	N1—C4—H4A	109.5
C3—N1—H1	108.3	C3^i^—C4—H4A	109.5
C4—N1—H1	108.3	N1—C4—H4B	109.5
O1—C1—O2	126.25 (16)	C3^i^—C4—H4B	109.5
O1—C1—C2	123.19 (15)	H4A—C4—H4B	108.1
O2—C1—C2	110.56 (14)	O3—P1—O5	116.28 (7)
N1—C2—C1	111.40 (13)	O3—P1—O4	109.25 (7)
N1—C2—H2A	109.3	O5—P1—O4	107.91 (7)
C1—C2—H2A	109.3	O3—P1—O6	109.29 (7)
N1—C2—H2B	109.3	O5—P1—O6	107.26 (7)
C1—C2—H2B	109.3	O4—P1—O6	106.41 (7)
H2A—C2—H2B	108.0	P1—O4—H4	109.5
N1—C3—C4^i^	110.32 (14)	P1—O6—H6	109.5
N1—C3—H3A	109.6	H1WA—O1W—H1WB	107.5
C4^i^—C3—H3A	109.6		

Symmetry codes: (i) −*x*+1, −*y*+1, −*z*.

### Hydrogen-bond geometry (Å, °)

**Table:**

*D*—H···*A*	*D*—H	H···*A*	*D*···*A*	*D*—H···*A*
O2—H2···O3	0.84	1.69	2.5324 (18)	177
N1—H1···O5^ii^	0.93	1.74	2.658 (2)	170
O4—H4···O1W	0.84	1.74	2.5729 (19)	172
O6—H6···O5^iii^	0.84	1.74	2.5700 (17)	169
O1W—H1WA···O6^iv^	0.85	2.08	2.868 (2)	155
O1W—H1WB···O3^v^	0.85	2.02	2.8633 (19)	169

Symmetry codes: (ii) −*x*+2, −*y*+1, −*z*+1; (iii) −*x*+2, *y*−1/2, −*z*+3/2; (iv) −*x*+2, −*y*+1, −*z*+2; (v) −*x*+2, *y*+1/2, −*z*+3/2.
